# Solvent-Free Microwave Extraction of Essential Oils from *Litsea cubeba* (Lour.) Pers. at Different Harvesting Times and Their Skin-Whitening Cosmetic Potential

**DOI:** 10.3390/antiox11122389

**Published:** 2022-12-01

**Authors:** Yufei Qiu, Yong Wang, Ying Li

**Affiliations:** 1 Guangdong International Joint Research Center for Oilseeds Biorefinery, Nutrition and Safety, Department of Food Science and Engineering, Jinan University, Guangzhou 510632, China; 2 Qingyuan Yaokang Biotechnology, Qingyuan 513200, China

**Keywords:** solvent-free microwave extraction, essential oil, *Litsea cubeba*, process optimization, harvesting time, cosmetic potential

## Abstract

*Litsea cubeba* fruit, which has the highest content of essential oils in the plant, is an important woody oil plant resource. In this study, the influence of the solvent-free microwave extraction (SFME) and hydrodistillation (HD) techniques on the extraction of *L. cubeba* fruit essential oils was investigated in terms of yield, kinetics, and chemical composition, where the former conditions were optimized by the response surface design. The maximal essential oil yield was obtained under the optimal SFME process conditions (442 W and 24 min), where the irradiation time was the most important variable (*p* < 0.0001). Regardless of the extraction method used, the influence of harvesting time on *L. cubeba* fruit essential oils were quantitatively and qualitatively analyzed afterwards, where the SFME essential oil from July showed its superiority over the others regarding its higher extraction yield and better bioactivities. Compared with the HD method, the SFME approach could significantly enhance the yield of essential oils extracted from June to August by nearly 47% with the advantages of saving energy and low environmental impact. Interestingly, the SFME method could selectively extract monoterpene hydrocarbons such as D-limonene with relation to different compositions and bioactivities. Moreover, SFME essential oil showed a better inhibitory effect on tyrosinase and melanogenesis, indicating its skin-whitening potential as a new promising natural cosmetic ingredient.

## 1. Introduction

China, with a growing population, is now faced with a shortage of oil supply due to the restriction of minimum arable land. Considering the abundant woody oil plant resources such as walnut, camellia, olive, and peony, these resources have recently become the alternative solution for increasing the self-sufficiency rate. Developing woody oil plants can not only effectively improve the ecological environment, but also provide a large number of feedstocks and various forest products. Most importantly, it may help to increase the income of farmers in poor mountainous areas.

*Litsea cubeba* (Lour.) Pers., one of special woody oil plants, is mainly distributed in the confined region of 20–30° N, 100–140° E that covers the Eastern Himalaya, Southern China and Southeast Asia [1]. Unlike other woody oil plants, *L. cubeba* is an economically important aromatic plant on account of its essential oil, with a lemon-like scent and pungent taste, which can be commercially extracted from the fruit, flowers, leaves, bark, and even roots by conventional hydrodistillation or steam distillation methods [2]. Although *L. cubeba* has been widely used in traditional medicine for a thousand years [3], its essential oil rather than kernel oil is still the main industrial export product with extensive use in the fields of food [4,5], medicine [6,7,8], cosmetics [9], and botanical insecticides [10]. China is the largest producer of *L. cubeba* essential oils in the world, with a total planting area of around 1440 thousand hectares, leading to about 140.1 thousand tons of essential oils, which is equivalent to nearly USD 0.14 billion. These *L. cubeba* essential oils were mostly exported to European and American countries for further purification and synthesis in value-added functional products.

*L. cubeba* essential oils from its different organs comprise various chemical constituents of varying proportions, where the pericarp of the fruit is considered to have the highest yield, accounting for 3–5%. This secondary metabolite has drawn widespread attention in recent years due to its biological activities as an antioxidant [11,12], anti-inflammatory [13], antimicrobial [14,15,16], anthelmintic [17,18], and anti-tumor [19]. Citral as the main chemical component in *L. cubeba* essential oil could be used as a natural synthon for the synthesis of high-grade fragrances (e.g., ionone, geraniol, citronellol, and even irone) and vitamin A and E as well [20,21]. Hence, it usually serves as an important recognized standard to evaluate the economic value of *L. cubeba* essential oils in international trade. Considering the level of citral accumulation in the *L. cubeba* essential oil, the harvesting time corresponding to fruit maturity has to be taken into account. Generally, the fresh fruit of *L. cubeba* collected in the summer for essential oil extraction should be before the mature period. Moreover, the period of changing fruit color from dark green (half-ripe) to purple black (overripe) is very short; thus, it is significant to know the appropriate harvesting time so as to guarantee the quality of the essential oils.

Apart from the harvesting time related to climate and geographic variations, the obtaining method is also one of important influencing factors for essential oils. Hydrodistillation (HD) is the most traditional method for obtaining essential oils from plants, which is composed of an external heating source, alembic, condenser, Florentine flask (separator), and cohobation system [22]. It usually leads to a relatively low extraction yield but with a long-time heating treatment, resulting in more energy consumption and the possibility of unexpected changes in aroma composition. These shortcomings call for innovative green extraction techniques to solve the problem, such as supercritical fluid, ultrasound, and microwave techniques [23,24,25]. Green extraction principles derived from the classic green chemistry and green engineering principles involve the use of renewable plant resources and alternative green solvents, production of by- and/or coproducts instead of wastes, and intensification or innovation of process procedures, which eventually obtains non-denatured and biodegradable extract/end-product without contaminants [26]. Solvent-free microwave extraction (SFME) is one of the emerging green extraction methods that has been proposed for the extraction of essential oil from aromatic herbs for years, which is a combination of microwave heating and dry distillation performed at atmospheric pressure without adding any solvents or water [27]. The internal water in plant cells will rapidly heat up and vaporize under microwave irradiation, causing the pressure in the intact cell to continuously rise, which causes the oleiferous glands to burst and collapse [25]. The SFME method makes full use of the in situ water in plant cells as a medium for extracting essential oils, which effectively avoids the interference of external media on the yield and quality of essential oils [28]. Moreover, unlike conventional HD, the directions of the mass transfer and heat conduction in the SFME process are both from the inside to the outside of the cells, which significantly accelerates the separation efficiency of essential oils [29].

In this study, the comparison of the SFME and HD extraction methods for essential oils from *L. cubeba* fruits at different harvesting times was conducted in terms of yield, kinetics, energy cost, and environmental impact, where the process conditions of the SFME method was first optimized by a face-centered, central composite design. Moreover, the influence of green SFME techniques on the chemical composition and in vitro antioxidant activity of *L. cubeba* essential oils at different harvesting times was investigated to further verify its consistency with green extraction principles. Furthermore, the inhibitory effect of *L. cubeba* essential oils on tyrosinase and melanogenesis was also investigated to verify their skin-whitening potential as natural cosmetic ingredients in the field of beauty and personal care.

## 2. Materials and Methods

### 2.1. Materials

Fresh *L. cubeba* fruits used for essential oil production were wild and collected during the harvesting time at the 15th day of June–August in 2021 by Qingyuan Yaokang Biotechnology (Qingyuan, China). Fresh plant materials that were not handled in time were temporarily stored in the refrigerator at 2–8 °C prior to extraction. The initial moisture content of fresh *L. cubeba* fruits was determined by an HE53 Moisture Analyzer (Mettler Toledo, Zurich, Switzerland).

The 2,2-Diphenyl-1-picrylhydrazyl (DPPH), randomly methylated-b-cyclodextrin (RMCD), 2,2′-azobis-amidinopropane (ABAP), dimethyl sulfoxide (DMSO), potassium hydroxide solution, acetone, ethanol, vitamin E, and L-tyrosine were purchased from Macklin in Shanghai, China, while the kojic acid and theophylline standards were obtained from GlpBio (Montclair, NJ, USA). The B16-F10 melanoma cells were obtained from Procell Life Science & Technology (Wuhan, China), and the dopa, tyrosinase from mushroom (≥1000 unit/mg of solid), and melanin standards were obtained from Sigma-Aldrich, Saint Louis, MO, USA. The TRIzol reagent was purchased from Invitrogen, Carlsbad, CA, USA; Dulbecco’s Modified Eagle Medium (DMEM), fetal bovine serum, penicillin/streptomycin, and trypsin-EDTA (0.25%) were obtained from Gibco, Grand Island, New York, NY, USA. The radio immunoprecipitation assay (RIPA) lysis buffer, Bradford dye-binding reagent, and cell counting kit-8 (CCK-8) were purchased from Beyotime, Shanghai, China. ChamQ Universal SYBR qPCR Master Mix was procured from Vazyme, Nanjing, China. All solvents and chemicals used were of an analytical grade.

### 2.2. Solvent-Free Microwave Extraction (SFME)

#### 2.2.1. Apparatus and Procedure

**Figure 1:**
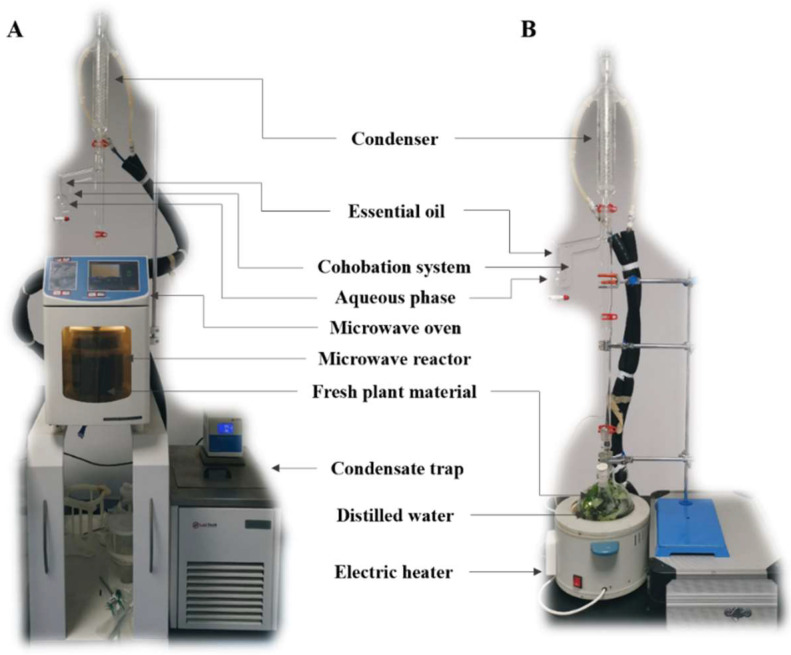
Apparatus for the (**A**) solvent-free microwave extraction and (**B**) conventional hydrodistillation.

As illustrated in Figure 1A, the SFME technique was performed in a NEOS-GR microwave apparatus (Milestone, Bergamo, Italy), which is a 2.45 GHz multimode microwave reactor with a controllable delivered power of up to 900 W. During extraction, the increase in the temperature in both the plant material and Pyrex glass extraction vessel was continuously monitored and recorded by embedded optical fiber sensors as a function of power. The SFME variables could be optimized for the maximization of the essential oil yield. Typically, the SFME method was performed at atmospheric pressure, where the plant essential oils and hydrolat entrained by the steam were condensed and eventually collected and separated by decantation. The extraction could be terminated when no more essential oil was extracted. At this moment, the essential oil was collected, dried with anhydrous sodium sulfate, and stored at 4 °C until subsequent analysis. The infrared probe installed in the SFME device could automatically monitor the real-time change in the temperature in the extraction vessel throughout the SFME process. Operators could easily notice and record the temperature displayed on the operation panel so as to avoid overheating and further possible thermal degradations. In addition to this built-in surveillance camera, the safety and quality control of extraction could also be guaranteed by an overheating warning program installed in the SFME device.

#### 2.2.2. Single-Factor Experiment

The effect of microwave irradiation power and time on the extraction yield of essential oil was firstly investigated in single-factor experiments. Fresh *L. cubeba* fruits (100 g) placed in the reactor were heated using different microwave irradiation powers (350, 400, 450, and 500 W) for 20 min and under different treatment times (10, 15, 20, and 25 min) when under a fixed microwave irradiation power of 450 W. After extractions, the yield of essential oil obtained was calculated as described below:

(1) $$\mathrm{Essential}\;\mathrm{oil}\;\mathrm{yield}\;\left(\%\right)=\frac{\mathrm{Weight}\;\mathrm{of}\;\mathrm{essential}\;\mathrm{oil}\;\mathrm{obtained}\;\mathrm{after}\;\mathrm{extraction}}{\mathrm{Weight}\;\mathrm{of}\;\mathrm{fresh}\;\mathrm{plant}\;\mathrm{materials}}\times 100$$

#### 2.2.3. Process Optimization—Face-Centered Central Composite Design (FCCD)

Based on the single-factor experimental results, two main factors and their appropriate ranges were finally established according to a three-level (−1, 0, +1) FCCD in order to investigate the relationship between the process variables and essential oil yield as the response value. Both the coded and actual values of independent variables are listed in Table 1, where the software Design Expert version 11.0 (Minneapolis, MN, USA), which included a built-in analysis of variance (ANOVA) tool was employed for the experimental design, data analysis, and modeling. The number of trials involved in FCCD is given by 2k + 2k + n, where k and n are the number of causal factors and center points, respectively. For the two variables, the design comprised 13 randomized experiments (k = 2 and n = 5) to avoid the effects of extraneous factors, with five repetitions at the center point to prove the suitability of the model. The fitted second-order polynomial model is as follows,

(2) $$Y=\beta_{0}+\overset{k}{\underset{i=1}{\sum }}\beta_{i}X_{i}+\overset{k}{\underset{i=1}{\sum }}\beta_{ii}X_{i}^{2}+\overset{}{\underset{i=1}{\sum }}\overset{}{\underset{j=i+j}{\sum }}\;\beta_{ij}X_{i}X_{j}$$

where *Y* represents the predicted essential oil yield, *X_i_* and *X_j_* represent the independent variables, k is the number of causal factors in SFME, and *β*_0_, *β_i_*, *β_ii_*, and *β_ij_* are the regression coefficients for the intercept, linearity, quadratic, and interactive terms, respectively.

**Table 1:** Experimental design matrix to screen the important variables for the extraction yield of *L. cubeba* fruit essential oil as the response.

Run	X_1_ Microwave Irradiation Power (W)	X_2_ Microwave Irradiation Time (min)	Response (Y) Essential Oil Yield (%)
1	450 (0)	20 (0)	1.800
2	450 (0)	20 (0)	1.710
3	450 (0)	20 (0)	1.800
4	400 (−1)	25 (+1)	1.710
5	400 (−1)	15 (−1)	0.945
6	500 (+1)	25 (+1)	1.620
7	500 (+1)	20 (0)	1.620
8	450 (0)	20 (0)	1.710
9	450 (0)	15 (−1)	1.260
10	400 (−1)	20 (0)	1.530
11	500 (+1)	15 (−1)	1.530
12	450 (0)	25 (+1)	1.800
13	450 (0)	20 (0)	1.620

The values in brackets are coded levels corresponding to real values from the single-factor experimental results.

### 2.3. Conventional Hydrodistillation (HD)

Until now, HD is still the mainstream method for the extraction of plant essential oil [7,30]. Fresh *L. cubeba* fruits (100 g) were mixed with 300 mL of distilled water for 1 h of extraction using a Clevenger-type apparatus (Figure 1B). In the HD method, plant materials were immersed in boiling water during long-term extraction. The resulting essential oil was collected, dried with anhydrous sodium sulfate, and stored at 4 °C for further analysis.

### 2.4. Kinetics of Essential Oil Extraction

The kinetics data of essential oils extracted from *L. cubeba* were fitted to the first-order rate equation derived from a quasi-steady model of extraction.

(3) $$In\left(Y_{\infty }/\left(Y_{\infty }-Y_{t}\right)\right)=k_{i}+a$$

where *Y_t_* is the yield of essential oil at time t, *Y_∞_* is the final yield of essential oil, k is the apparent first-order rate constant of extraction, and a is the semi-empirical intercept. $In\left(Y_{\infty }/\left(Y_{\infty }-Y_{t}\right)\right)$ plotting against time results in two intersecting straight lines, where the first is a relatively steep slope and another slope is a relatively shallow one.

### 2.5. Scanning Electron Microscope (SEM)

In order to verify the integrity of essential oil glands, *L. cubeba* peels were carefully collected from its fruits before and after the SFME and HD techniques and directly gold-plated to provide electrical conductivity before being observed by scanning electron microscopy (Zeiss Merlin, Oberkochen, Germany).

### 2.6. Gas Chromatography-Mass Spectrometry (GC-MS) Analysis

A GC-MS analysis was performed using a Hewlett Packard 7890 gas chromatograph interfaced with a Hewlett Packard mass spectrometer 5975 (Agilent Technologies, Palo Alto, CA, USA) equipped with an HP-5MS capillary column (15 m × 0.25 mm, i.d., 0.25 μm film thicknesses). The column temperature was held at 50 °C and increased from 50 °C to 60 °C at a rate of 2 °C/min, being held at 60 °C for 4 min. Subsequently, the temperature increased from 60 °C to 160 °C at a rate of 2.5 °C/min and was held at 160 °C for 2 min. Finally, the temperature increased from 160 °C to 285 °C at a rate of 25 °C/min and was held at 285 °C for 1 min. For GC-MS detection, an electron ionization system with an ionization energy of 70 eV was used over a scan range of 50–500 m/z. Helium gas was used as the carrier gas at a constant flow rate of 1 mL/min in a split mode of 1:50 with an injection volume of 1 μL. Injector and detector temperatures were both 280 °C. The ion source temperature was 280 °C. The components were identified by matching their recorded mass spectra with the mass spectra data bank (NIST14 Library) combined with a comparison of their GC retention indices relative to a series of n-hydrocarbons (C7–C40).

### 2.7. In Vitro Antioxidant Activity

#### 2.7.1. 2,2-Diphenyl-1-picrylhydrazyl (DPPH) Radical Scavenging Assay

The in vitro antioxidant activity of *L. cubeba* essential oil was firstly evaluated by testing the scavenging ability of DPPH (2,2-diphenyl-1-picrylhydrazyl), which is a stable highly colored free radical that can turn to a colorless hydrazine by abstracting hydrogen atoms from phenolic antioxidants [31]. Briefly, a DPPH solution in ethanol (0.2 mmol/L) was prepared, and 160 μL of the DPPH solution and 80 μL of the essential oil at different concentrations were thoroughly mixed and stored at room temperature for 30 min. The absorbance was measured at 517 nm with ethanol as the blank. The capacity of scavenging the DPPH radical was expressed as the percentage of reduced DPPH, calculated by the following equation:

(4) $$\mathrm{DPPH}\;\mathrm{scavenging}\;\mathrm{activity}\;\left(\%\right)=\left[1-\frac{A_{\mathrm{sample}}-A_{\mathrm{blank}}}{A_{\mathrm{control}}}\right]\times 100$$

where A_sample_ is the absorbance of ethanol solutions with DPPH and samples, A_blank_ is the absorbance of ethanol solutions with only samples, and A_control_ is the absorbance of ethanol solutions with only DPPH. All experiments were performed in triplicates.

#### 2.7.2. Peroxyl Radical Scavenging Capacity (PSC) Assay

The PSC assay was determined according to the method of Adom and Liu [32] with a slight modification. The essential oil was dissolved in 12% RMCD prepared in 50% of acetone in water. A VE standard or sample of 100 μL was blended with 100 μL of oxidize nonfluorescent dichloroflucorescein (DCFH), which was obtained by hydrolyzing 2.48 mM of dichlorofluorescin diacetate (DCFH-DA) with 1 mmol/L of KOH. Subsequently, 50 μL of ABAP (300 mM) was added to initiate the reaction at 37 °C. The fluorescence of the mixture at 485 nm excitation and 538 nm emission was measured every 2 min in a 45 min run. The areas under the average fluorescence reaction time kinetic curve (AUC) for both the control and samples were integrated.

(5) $$\mathrm{PSC}\;\mathrm{unit}=1-\frac{SA}{\mathrm{CA}}$$

where *SA* is the AUC for the sample or standard dilution and CA is the AUC for the control reaction using only 12% RMCD. The median effective concentration (EC_50_) was defined as the dose required to cause a 50% inhibition (PSC unit = 0.5) for each sample extracted, which was also used as the basis for comparing different samples. More powerful antioxidants would have lower EC_50_ values. The PSC value is the EC_50_ expressed in μmol of vitamin E equivalent per gram of sample (μmol VE/g), which was used as the in vitro antioxidant activity of the *L. cubeba* essential oils. All experiments were performed in triplicates.

### 2.8. Tyrosinase Inhibition Assay

A tyrosinase inhibition assay was performed according to the modified dopachrome method using L-tyrosine as a substrate [33]. The essential oil and control sample of 40 μL mixed with 100 μL of tyrosinase (250 units/mL in 0.1 M of phosphate buffer, pH 6.8) was incubated for 10 min. The reaction was then initiated by adding 100 μL of L-tyrosine (3 mM). After 10 min of incubation, the absorbance was taken at 475 nm using a 96-well microplate reader. The assay was performed in triplicate, and the inhibitory effect was calculated in comparison with kojic acid. The percent inhibition of tyrosinase enzyme was calculated using Equation (6) and when the IC_50_ values showed more than 50% inhibition from the dose-effect curves.

(6) $$\mathrm{Inhibition}\;\left(\%\right)=\left[\frac{A_{\mathrm{control}}-A_{\mathrm{sample}}}{A_{\mathrm{control}}}\right]\times 100$$

where A_sample_ is the absorbance of the sample extracts and A_control_ is the absorbance of the blank control using the buffer instead of the inhibitor (sample).

### 2.9. Melanogenesis Inhibition Assay

#### 2.9.1. Cell Culture

B16-F10 melanoma cells were cultured in a 25 cm^2^ flask in DMEM medium supplemented with 10% fetal bovine serum and 1% penicillin/streptomycin at 37 °C in a humidified incubator with 5% CO_2_. Cells were grown to semi-confluence and harvested by 0.25% trypsin (*w*/*v*) and 0.06 mM of EDTA in phosphate buffer saline. All experiments in the cell culture were performed in triplicate.

#### 2.9.2. Cytotoxicity Assay

The CCK-8 (cell counting kit 8) assay was used for cell cytotoxicity determination [34]. Cells (1 × 10^4^ cells/well) were incubated in a 96-well plate for 24 h and then treated with different concentrations of the samples for 24 h. Subsequently, 10 μL of CCK-8 solution was added to each well, which was placed in an incubator at 37 °C and humidified with 5% CO_2_ for 2 h. The absorbance at 450 nm was measured with a microplate reader. The cell viability was compared with the control treated with 0.45% DMSO, which percentage was calculated as Equation (7) describes,

(7) $$\mathrm{Cell}\;\mathrm{viability}\;\left(\%\right)=\left(\frac{\mathrm{Absorbance}\;\mathrm{of}\;\mathrm{sample}}{\mathrm{Absorbance}\;\mathrm{of}\;\mathrm{control}}\right)\times 100$$

#### 2.9.3. Melanin Content Measurement

The melanin content was measured according to the previously described method with a slight modification [35]. Briefly, B16-F10 melanoma cells at a density of 1 × 10^5^ cells/well were plated in 6-well plates and incubated overnight for cell adhesion. Samples at different concentrations, kojic acid (positive control), and theophylline (negative control) were then added and incubated for 72 h. Cells were harvested by 0.25% trypsin (*w*/*v*) and 0.06 mM EDTA in phosphate buffer saline. The cells were washed and dissolved in 500 μL of 2 M sodium hydroxide containing 10% DMSO at 60 °C for 1 h. The absorbance was measured at 450 nm using a microplate reader, and the melanin content was compared to the standard melanin. The total protein content of cell lysate was also evaluated by the Bradford dye-binding reagent using bovine serum albumin as a standard, and the absorption was measured at 595 nm. For the determination of the actual melanin formation from the same cell concentration, the melanin content of each treatment was divided by the total protein content. The percentage of the relative ratio of melanin content was calculated as Equation (8) describes,

(8) $$\mathrm{Melanin}\;\mathrm{content}\;\left(\%\right)=\left(\frac{\mathrm{Melanin}\;\mathrm{content}\;\mathrm{of}\;\mathrm{sample}\;/\;\mathrm{total}\;\mathrm{protein}\;\mathrm{content}\;\mathrm{of}\;\mathrm{sample}}{\mathrm{Melanin}\;\mathrm{content}\;\mathrm{of}\;\mathrm{control}\;/\;\mathrm{total}\;\mathrm{protein}\;\mathrm{content}\;\mathrm{of}\;\mathrm{control}}\right)\times 100$$

#### 2.9.4. Tyrosinase Activity Assessment

Tyrosinase activity was analyzed by the previously described method with a slight modification [36]. Briefly, B16-F10 melanoma (1 × 10^5^ cells/well) incubated in 6-well plates for 24 h was treated with the samples and incubated for 48 h. The treated cells were washed, lysed with RIPA buffer containing protease inhibitor, and then incubated at 4 °C for 30 min before a centrifugation at 14,000 rpm for 10 min. The obtained supernatant was collected, mixed with the mixture containing 50 mM of sodium phosphate buffer (pH 6.8) and 0.05% dopa, and further incubated at 37 °C for 2 h. After incubation, the dopachrome formation was measured at 490 nm using a microplate reader. The enzyme activity was calculated compared with the standard mushroom tyrosinase. The total protein content of sample was also evaluated. The percentage of the relative ratio of tyrosinase activity was calculated using Equation (9) shown as follows,

(9) $$\mathrm{Tyrosinase}\;\mathrm{activity}\;\left(\%\right)=\left(\frac{\mathrm{Tyrosinase}\;\mathrm{activity}\;\mathrm{of}\;\mathrm{sample}\;/\;\mathrm{total}\;\mathrm{protein}\;\mathrm{content}\;\mathrm{of}\;\mathrm{sample}}{\mathrm{Tyrosinase}\;\mathrm{activity}\;\mathrm{of}\;\mathrm{control}\;/\;\mathrm{total}\;\mathrm{protein}\;\mathrm{content}\;\mathrm{of}\;\mathrm{control}}\right)\times 100$$

#### 2.9.5. Tyrosinase Related Proteins-2 (TRP-2) Gene Expression

The gene expression of TRP-2 was determined by RT-qPCR analysis [37]. B16-F10 melanoma cells plated in a 6-well plate were incubated overnight at a density of 1 × 10^6^ cells/well. The attached cells were treated with different concentrations of samples for 24 h and then collected for the extraction of total RNAs using TRIzol reagent, where 1 μg of total RNA was subsequently used to generate cDNA using an RT-PCR system. Target gene amplification was performed using specific oligonucleotide primers in a normal PCR system. The primer sequences were as follows: TRP-2, forward (5′-GCAAGAGATACACGGAGGAAG-3′), and reverse (5′-CTAAGGCATCATCATCATCACTAC-3′); β-actin, forward (5′-AGGCCAACCGTGAAAAGATG-3′), and reverse (5′-ATGCCAGTGGTACGACCAGA-3′). The qPCR was conducted using a ChamQ Universal SYBR qPCR Master Mix reagent kit (Vazyme, Nanjing, China) according to the manufacturer’s instructions. Gene expressions were performed in triplicates, and the mean of these values was used for analysis. The relative gene expression level was calculated by the 2^−ΔΔCT^ method.

### 2.10. Statistical Analysis

The statistical analysis of variance (ANOVA) was determined using R software (version 3.6.3) with Tukey’s test with a significant difference at the 95% level between samples. Significant differences (*p* < 0.05) shown by each sample tested were labeled as different superscript patterns. Data were presented as means ± standard deviation. All experiments were performed in at least triplicates.

## 3. Results and Discussion

### 3.1. The Effect of Microwave Irradiation Power and Time on SFME Yield

**Figure 2:**
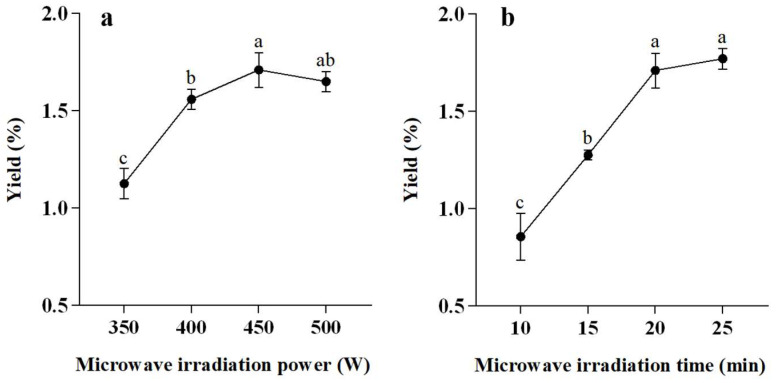
The effect of microwave irradiation (**a**) power and (**b**) time on the yield of *L. cubeba* essential oil. Yield with the same letter are not significant at *p* < 0.05, which are presented as the means ± standard deviation of the triplicate.

High microwave irradiation power generally results in high temperature in the extraction system [38]. Since the real-time monitoring of temperature change in the SFME vessel could help to ensure that the quality of extraction, single-factor experiments for microwave irradiation power, and time were conducted before subsequent parameter optimization design. To investigate the effect of the microwave irradiation power on the SFME yield of *L. cubeba* essential oil, extractions were carried out with a fixed time of 20 min at 350, 400, 450 and 500 W, respectively. As Figure 2a shows, the extraction yield of essential oil was significantly influenced when the microwave power was less than 450 W, whereas it slightly decreased when the microwave power was greater than 450 W. As previously reported, the application of high microwave irradiation power resulted in the overheating and carbonization of plant materials and thus led to a low extraction yield [39]. Similarly, the effect of microwave irradiation time (10, 15, 20 and 25 min) in the SFME method was successively studied using a fixed microwave power of 450 W (Figure 2b). The extraction yield of *L. cubeba* essential oil dramatically increased within the first 20 min and gradually reached equilibrium after 20 min. Therefore, considering the high extraction yield and low energy consumption, microwave power range of 400–500 W, and extraction time range of 15–25 min, were selected for further optimization design experiments.

### 3.2. SFME Optimization Design

#### 3.2.1. Model Fitting and Analysis

For the experimental design, microwave irradiation power (X_1_) and time (X_2_) as two independent variables were evaluated by the FCCD to study their effects on the extraction yield of *L. cubeba* essential oil (Y). This design has three levels with axial points embedded in the fact centers combining with center points. As Table 1 shows, the microwave irradiation power and time were set at low (coded level: −1), intermediate (coded level: 0), and high (coded level: +1) levels based on preliminary single-factor experimental results. The FCCD results showed a wide variation in the extraction efficiency of essential oil, which reflected the importance of process optimization to attain a higher yield of essential oil. In order to further study the interactions between two factors, their effects on the extraction yield of essential oil were presented in Table 2. ANOVA and regression analyses were used to test the adequacy and goodness-of-fit of the quadratic model equation. For the high F-value (24.12) of the model in this FCCD, there was only a 0.03% probability (*p*-value) of error from noise, indicating the significance of the model chosen. A lower *p*-value (*p* < 0.0001) demonstrated an extremely high significance and good fitting of the proposed model. The lack of fit (0.9576) was not significant as evidenced by a relatively high *p*-value (*p* > 0.05), which indicated that the adopted quadratic model was well-fitted with better credibility and accuracy. The adequacy and goodness of fit between the predicted values and the actual experimental results of essential oil yields can be further demonstrated using the R^2^ and R^2^_Adj_ values obtained by regression analysis. The R^2^ value of 0.9451 showed that at least 94% of actual experimental results matched the model while the R^2^_Adj_ value of 0.9059 indicated a high correlation between actual experimental results and predicted values. The precision was high with decent experimental results due to the low coefficient of variation (C.V. = 4.70%).

The relationship between the extraction yield of essential oil and two SFME operation parameters in terms of coded levels could be explained by the following equation. Because the response in this FCCD only contained one factor, a logical interpretation for this proposed model is that all terms including linear, interaction, and quadratic coefficients significantly affected the extraction yield of essential oil (*p* < 0.05).

Y = 1.72 + 0.0975X_1_ + 0.2325X_2_ − 0.1687X_1_X_2_ − 0.1172X_1_^2^ − 0.1622X_2_^2^ (10)

#### 3.2.2. Response Surface Analysis

**Figure 3:**
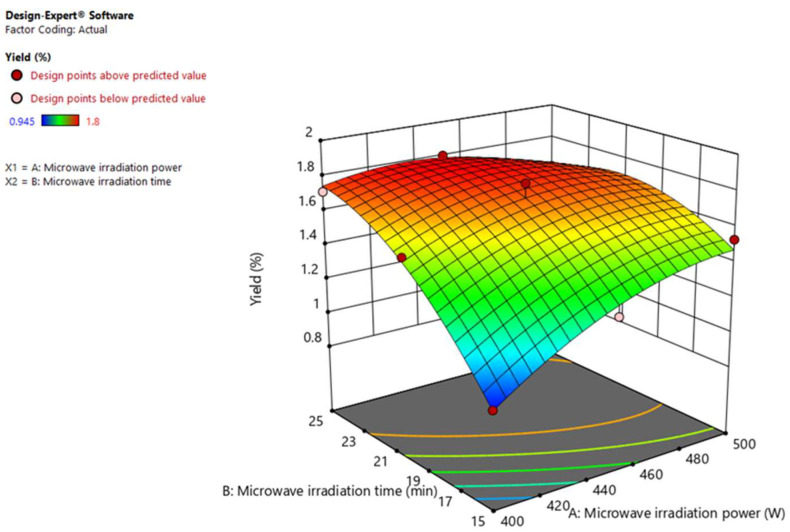
Three-dimensional response surface plots for the effect of microwave irradiation power and time on the yield of *L. cubeba* fruit essential oil.

A three-dimensional response surface plot was established to explain the effect of two independent variables on the extraction yield of essential oils. The dark red color indicates the highest yield of essential oil, and the dark blue color indicates the lowest yield (Figure 3). Microwave irradiation power and time showed their significant effects on the extraction yield of *L. cubeba* essential oil in a linear, interacted, and quadratic manner, which could be attributed to the acceleration of mass and heat transfer from plant cells inside to the external environment by microwave irradiation power. The result was consistent with previous studies [27,39], where microwave irradiation power and time also showed a positive effect on the essential oil yield. The maximum yields of essential oils could be obtained at a microwave power of around 450 W and irradiation time of 20–25 min. Nonetheless, it should be noted that overheating may cause the decomposition of thermolabile components in essential oils.

#### 3.2.3. Experimental Validation of Predictive Model

The optimal extraction conditions of the SFME procedure were determined as a microwave power of 441.94 W and irradiation time of 24.01 min for maximizing the extraction yield of *L. cubeba* essential oils. For the high feasibility in actual operations, the predicted optimal conditions from FCCD were adjusted to a microwave power of 442 W and irradiation time of 24 min, which brought about 1.80 ± 0.08% of extraction yield. Therefore, the accuracy and acceptability of the response model for SFME optimization was proven by the good correlation between the experimental and predicted values.

### 3.3. Extraction Kinetics Comparison of SFME and HD

**Figure 4:**
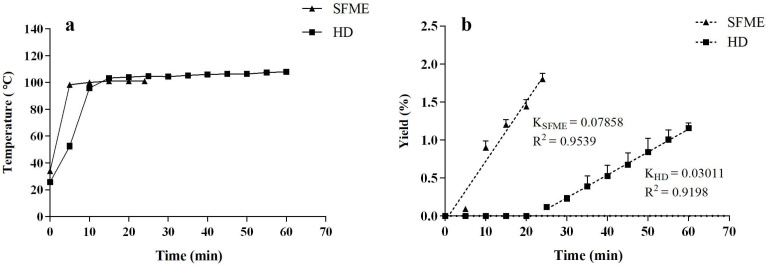
Comparison of extraction (**a**) temperature and (**b**) kinetics between solvent-free microwave extraction (SFME) and hydrodistillation (HD) processes.

Kinetic modeling is an approach for understanding the heating conduction and mass transfer behaviors in the SFME and HD processes used for *L. cubeba* essential oil extraction. The variation in essential oil yield and extraction temperature as a function of the extraction time during the SFME and HD processes was plotted in Figure 4. For conventional heating in the HD method, the extraction temperature recorded was closely related to the temperature of the water surrounding the plant material, whereas that in the SFME approach was more relevant to the limited in situ water in the plant material. It is worth mentioning that HD required nearly 13 min to reach the boiling point of water (100 °C), whereas the optimized SFME at 442 W could approach 100 °C in only 5 min (Figure 4a). The entire SFME process could obtain 1.80 ± 0.08% of *L. cubeba* essential oil yield within just 24 min compared with the 1.16 ± 0.07% yielded by HD in 60 min. Because microwave heating is the only variable modified between both experiments, it clearly improved the kinetics of extraction. For both techniques, the extraction of essential oils that occurred in their distinct kinetic curves was demonstrated by the rupture of the slope in the linear plots (Figure 4b). The first-order kinetic model was well adopted for both extraction procedures with high R^2^ values (0.9539 for SFME and 0.9198 for HD). Because the extraction rate constant (K) for SFME (0.07858) was more than twice as high as that of HD (0.03011), it can be concluded that microwave irradiation heating could significantly modify the extraction kinetics, resulting in a reduced extraction time compared with the HD method. However, this finding was not in accordance with other green extraction techniques such as ultrasound, though ultrasound could largely enhance the extraction yield similarly to microwave approaches [40]. The extraction kinetics and essential oil yields in the SFME method might also be affected by the steam flow rate and type of microwave devices, which should also be taken into consideration for further scaling-up designs.

### 3.4. Impact of Microwave on Microstructure

**Figure 5:**
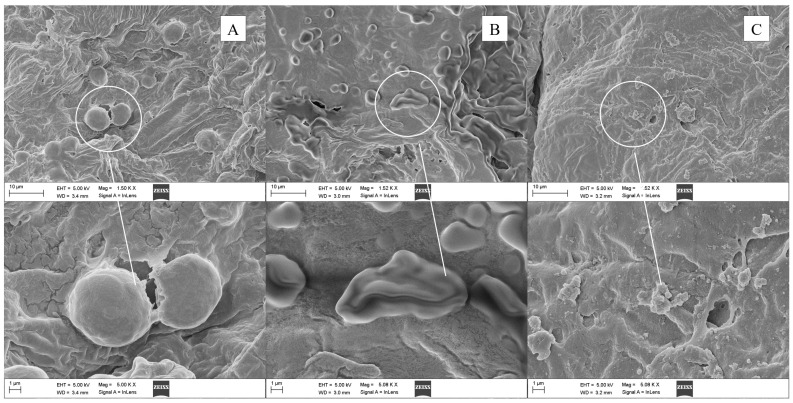
The upper epidermis of *L. cubeba* fresh fruits observed by SEM: (**A**) before extraction; (**B**) after hydrodistillation; and (**C**) after solvent-free microwave extraction.

Because different extraction methods produced distinguishable morphological changes in plant cell tissues, an SEM analysis was performed on *L. cubeba* fruits in order to investigate the effects of the SFME and HD methods on the solid matrix. Before extraction, *L. cubeba* essential oil glands appeared to be relatively intact and full at the upper epidermis of the fresh *L. cubeba* fruits (Figure 5A). After the HD process, these glands dehydrated and released part of their contents, but most of them remained intact (Figure 5B), indicating that HD was not sufficient to obtain a complete extraction despite its long treatment time. The intact cell wall and wrinkled outer surface structure could impede the diffusion of intracellular volatiles. Nevertheless, in the case of the SFME approach, these oil glands were subjected to more severe thermal stresses and localized high pressures induced by microwave heating, resulting in cell rupture and easier crumbling [41]. Thus, only cellular debris could be found in Figure 5C, which demonstrated the efficiency of the SFME method corresponding to its higher extraction yield compared with the HD technique. 

### 3.5. Cost, Cleanliness, Up-Scaling, and Safety Considerations

Compared with conventional the HD method, the lower cost and environmental impact are great advantages for the SFME approach in terms of time, energy, and solvent consumption. The power consumption was determined with a wattmeter at the microwave generator entrance and electrical heater power supply. The conventional HD process required an extraction time of 60 min, corresponding to 347.96 ± 20.57 kWh/kg essential oil; SFME only required 24 min, corresponding to 166.33 ± 7.50 kWh/kg essential oil. The environmental impact of the SFME and HD methods was estimated by calculating the quantity of the CO_2_ rejected in the atmosphere. As previously reported, 800 g of CO_2_ will be released into the atmosphere by the combustion of fossil fuels during the process of generating 1 kWh of electricity [42]. Thus, the mass of CO_2_ produced during the traditional HD method (278.37 ± 16.46 kg/kg essential oil) was much larger than that generated from the SFME process (133.06 ± 6.00 kg/kg essential oil). Moreover, large-scale SFME processing has also been commercially available with a comparable yield to the lab scale experiments for essential oils from aromatic herbs [28]. Apart from the achievable benefits from SFME such as low maintenance cost and improvements in process efficiency and product quality, waste disposal could be much easier to handle due to the fact that the peel of fresh *L. cubeba* could be completely removed after SFME to obtain the pure kernels for further oil pressing as the value-added medium-chain saturated fatty acid feedstock [43,44].

### 3.6. Impact of Extraction Methods on Chemical Composition of L. cubeba Essential Oils at Different Harvesting Times

Table 3 lists the yield and grouped compounds of *L. cubeba* fruit essential oil from both extraction methods at different harvesting times, accounting for 99.03–99.71% of the total essential oil. Regardless of the extraction method used, the main component identified in *L. cubeba* fruit essential oils was geranial (α-citral), neral (β-citral) and D-limonene, which is consistent with previous studies [45,46]. Concerning the composition of *L. cubeba* fruit essential oil, it was found that harvesting time indeed had a notable influencing factor on both extraction types. During the harvesting time, the content of citral gradually accumulated in *L. cubeba* fruits from June to August while the content of D-limonene was dramatically decreased, which is also consistent with previous studies [12]. Compared to the HD technique using a large quantity of water and longer heating time, the SFME method generally required less intense thermal and hydrolytic effects, which could selectively extract more D-limonene instead of citral. Additionally, the content of minor components such as 3-carene, β-caryophyllene, isogeranial, isoneral, and citronellal was also significantly increased in SFME essential oil, indicating the higher selectivity and superiority of microwave heating for retaining the valuable aromatic bioactive compounds. In total, substantial amounts of oxygenated compounds (80.18–90.44% vs. 65.37–80.28%) and lower amounts of monoterpene hydrocarbons (8.3–17.99% vs. 18.52–33.06%) were present in *L. cubeba* fruit essential oils extracted by the HD method in comparison with the SFME approach. Although oxygenated compounds with a high dipolar moment are theoretically more easily extracted by microwave methods [28], the higher proportion of non-oxygenated compounds was found in this study using SFME, which is mainly attributed to the selective extraction of D-limonene.

In light of the extraction yield of essential oils at different harvesting times, the SFME method could significantly increase the extraction yield of *L. cubeba* fruit essential oils compared to the HD approach. The extraction yield was highest in July regardless of the extraction method used, indicating that the maturity of *L. cubeba* fruits in July was the best for its essential oil extraction. After August, *L. cubeba* fruits will be overripe, resulting in the degradation of volatile components and the difficulty of picking and collection. Considering both yield and quality, *L. cubeba* fruit essential oils from July using SFME under optimized conditions were selected for further bioactivity evaluations.

### 3.7. In Vitro Antioxidant Activities

Because a natural plant extract contains multiple components, its antioxidant capacity is usually ascertained by at least two or more methods. In this study, the antioxidant activity of *L. cubeba* fruit essential oils from HD and SFME at different harvesting times was studied by two in vitro radical scavenging assays related to DPPH and peroxyl radicals [47]. The IC_50_ value (i.e., half maximal inhibitory concentration) was used to evaluate the antioxidant capacity of essential oils in the DPPH radical scavenging assay, while vitamin E was used as the equivalent to compare the antioxidant activity in the PSC assay.

**Figure 6:**
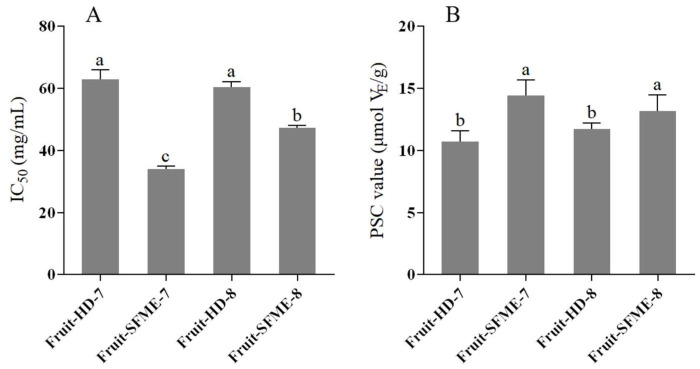
In vitro antioxidant activity of *L. cubeba* fruit essential oils from July (7) and August (8) obtained by hydrodistillation (HD) and solvent-free microwave extraction (SFME) techniques: (**A**) DPPH radical scavenging assay and (**B**) peroxyl radical scavenging capacity (PSC) assay. Columns with the different letter are significantly different at *p* < 0.05.

The initial moisture content of *L. cubeba* fruits at the July and August periods was measured as 68.99 ± 1.48% and 73.05 ± 2.59%, respectively. As Figure 6 depicts, it was found that similar results were obtained from both DPPH and PSC assays, where SFME essential oils showed better antioxidant activities than HD essential oils. In the DPPH radical scavenging assay, HD essential oils from July and August displayed an insignificant difference in their antioxidant activities, whereas the opposite result was obtained for SFME essential oils (Figure 6A). In the PSC assay, the harvesting time demonstrated no influence on the antioxidant activity of essential oils from the same extraction method (Figure 6B). Overall, it was worthwhile to note that SFME essential oils from July showed the strongest antioxidant activity with the lowest IC_50_ value (34.04 mg/mL) and highest PSC value (14.39 μmol VE/g), which was probably related to the change in the chemical composition caused by the selectivity of SFME and the maturity of the *L. cubeba* fruits. Hence, SFME essential oil from July stood out as the candidate for the further investigations of its skin-whitening cosmetic potential.

### 3.8. Tyrosinase Inhibitory Activities

Tyrosinase is a rate-limiting enzyme which can catalyze both tyrosine and dihydroxy phenylalanine (dopa) as two substrates in the first two steps of melanogenesis process. Mushroom tyrosinase has extensively been used as a key enzyme to test melanogenesis inhibitors [48]. The inhibitory effect of *L. cubeba* essential oils on tyrosinase activity was investigated. As compared to the positive standard kojic acid, both the SFME and HD essential oils revealed considerable inhibitory activities against tyrosinase because of the presence of citral and D-limonene as tyrosinase inhibitors [9]; SFME essential oil showed a significantly higher tyrosinase inhibitory activity (IC_50_ 13.10 ± 0.25 mg/mL) than that of HD essential oil (IC_50_ 16.64 ± 0.37 mg/mL).

### 3.9. Melanogenesis Assay in B16-F10 Melanoma Cells

Skin hyperpigmentation has been reported as one of the greatest concerns in cosmetic, beauty, and dermatologic fields. For years, the discovery of safe natural skin-whitening agents has never been stopped [35]. Melanogenesis involving three major melanogenic enzymes (i.e., tyrosinase, TRP-1, and TRP-2) is a complicated pigment biosynthesis process in melanocytes [49]. For pigment formation, melanoma cells have been demonstrated to have similar results to normal melanocytes for cell proliferation and in vitro assays. Thus, the B16-F10 melanoma cell line was selected for use in the melanogenesis assay.

**Figure 7:**
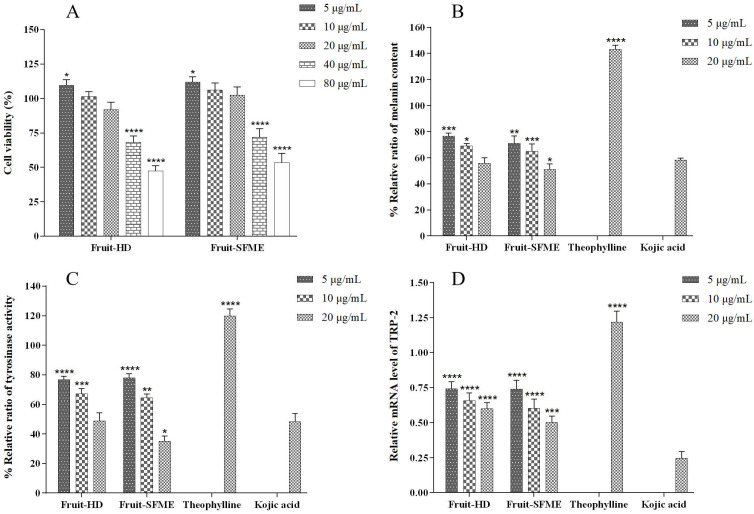
Melanogenesis assay in B16-F10 melanoma cells: (**A**) cytotoxicity assay in B16-F10 melanoma cells treated with *L. cubeba* fruit essential oils at 5–80 μg/mL. The asterisk * indicates a significant difference from the control (* *p* < 0.05, **** *p* < 0.0001); (**B**) percentage of relative ratio of melanin content, (**C**) tyrosinase activity, and (**D**) relative mRNA level of TRP-2 in B16-F10 melanoma cells treated with essential oils, theophylline, and kojic acid at 5–20 μg/mL. The asterisk * indicates a significant difference from the kojic acid (* *p* < 0.05, ** *p* < 0.01, *** *p* < 0.001, and **** *p* < 0.0001).

The cytotoxicity assay of *L. cubeba* fruit essential oil was studied in B16-F10 melanoma cells to obtain the non-cytotoxic concentrations for further analysis in the melanogenesis assay. As shown in Figure 7A, the cytotoxicity effect of *L. cubeba* essential oil was concentration dependent. For B16-F10 melanoma cells, a cell viability of greater than 80% was observed in the cells treated with essential oils at 5–20 μg/mL, indicating the non-cytotoxic effect. The further increase in the concentration of both HD and SFME essential oil decreased the cell viability, where essential oils at 80 μg/mL resulted in the decline in cell viability to the minimum for both HD (47.33 ± 3.13%) and SFME (53.48 ± 5.31%) essential oils.

The non-cytotoxic concentrations of *L. cubeba* essential oil were further used to evaluate the anti-melanogenesis effect in B16–F10 melanoma cells. Theophylline (pigment stimulator) and kojic acid (pigment inhibitor) at the non-cytotoxic concentration of 20 μg/mL were employed as the positive and negative controls, respectively. The percentage of the relative ratio of melanin content, tyrosinase activity, and relative mRNA level of TRP-2 in B16F10 melanoma cells treated with various concentrations of samples is illustrated in Figure 7. The inhibitory effect of *L. cubeba* essential oil on melanin content appeared to be in the concentration-dependent manner as well (Figure 7B). A decreased melanin content was observed with the increase in concentration of *L. cubeba* essential oils from 5–20 μg/mL. The percentages of melanin content for theophylline and kojic acid were 143.14 ± 2.47 and 58.32 ± 1.08%, respectively. Although no significant difference was found in the inhibitory activity of *L. cubeba* essential oil and kojic acid on melanin formation, the inhibitory activity of SFME essential oil from July at 20 μg/mL was significantly higher than that of HD essential oil with a relative ratio of melanin content of 50.99 ± 3.34%.

The tyrosinase activities and TRP-2 mRNA expression of cells treated with *L. cubeba* essential oil, theophylline, and kojic acid were correlated with the effect on melanin content (Figure 7C,D). The enzymatic activities of melanogenesis in cells treated with theophylline and kojic acid were related to their effects on melanin formation [35]. Theophylline showed an increased activity of the tyrosinase and mRNA level of TRP-2 to 119.88 ± 3.77% and 1.22 ± 0.06, respectively. On the contrary, the tyrosinase activity and TRP-2 mRNA level for kojic acid were 48.39 ± 4.35% and 0.25 ± 0.04, respectively. The significant decrease in the tyrosinase activity and mRNA level of TRP-2 was observed in samples treated with *L. cubeba* essential oil at 5, 10, and 20 μg/mL. HD and SFME essential oils from July appeared to significantly decrease the tyrosinase activity to 48.94 ± 4.42% and 35.17 ± 2.79%, respectively, which corresponded to their mRNA levels of TRP-2 (0.60 ± 0.03, 0.50 ± 0.04). It was notable that the decreased melanin content and melanogenic enzyme activities of cells treated with SFME essential oil from July demonstrated a significantly better inhibitory effect on the melanogenesis process than that of HD essential oil.

## 4. Conclusions

Based on the results above, the SFME extraction of *L. cubeba* fruit essential oil in line with green extraction principles has proved its superiority to the conventional HD method. Firstly, the SFME technique heated the in situ water in plant cells without adding external water, avoiding a large amount of solvent use and the possibility of hydrolytic degradation. Secondly, the same direction of mass and heat transfer in SFME led to a rapid heating, which significantly improved the extraction efficiency with reduced time and energy consumption corresponding to a lower production cost and environmental impact. Furthermore, the chemical composition of *L. cubeba* fruit essential oil could be influenced by both the extraction method and harvesting time, which should also be considered for further scaling-up and quality control. Last but not least, SFME essential oil harvested from July performed the best with respect to both yield and antioxidant capacity and also demonstrated the best anti-melanogenesis effect. Given this, SFME is a green, cleaner, eco-friendly, and efficient technique that is suitable for extracting essential oils from *L. cubeba* fruits, and has shown great potential as an alternative to the conventional HD approach. The SFME essential oils harvested from July proved their potency as functional ingredients in cosmetic formulations and the beauty care field.

## Figures and Tables

**Table 2:** Analysis of variance (ANOVA) for the experimental results in the optimization design.

Source	Sum of Squares	Degree of Freedom	Mean Square	F-Value	*p*-Value	
Model	0.6713	5	0.1343	24.12	0.0003	Highly significant
X_1_	0.0570	1	0.0570	10.25	0.0150	
X_2_	0.3243	1	0.3243	58.26	0.0001	
X_1_X_2_	0.1139	1	0.1139	20.46	0.0027	
X_1_^2^	0.0379	1	0.0379	6.81	0.0349	
X_2_^2^	0.0726	1	0.0726	13.04	0.0086	
Residual	0.0390	7	0.0056			
Lack of fit	0.0163	3	0.0054	0.9576	0.4938	Not significant
Pure error	0.0227	4	0.0057			
Cor total ^a^	0.7103	12				
R^2^	0.9451					
R^2^_Adj_	0.9059					
C.V. ^b^%	4.70					

X_1_: Microwave irradiation power (W), X_2_: Microwave irradiation time (min). ^a^ Totals of all information corrected for the mean. ^b^ Coefficient of variation.

**Table 3:** Chemical composition of *L. cubeba* fruit essential oils from hydrodistillation (HD) and solvent-free microwave extraction (SFME) at different harvesting times.

Peak	RI ^#^	Compounds	Relative Percentage (%)					
			HD—June	HD—July	HD— August	SFME—June	SFME—July	SFME—August
		Monoterpenes						
1	928	3-Thujene	0.05	0.04	0.03	0.05	0.05	0.03
2	934	3-Carene	0.80	0.58	0.56	1.68	1.43	1.09
3	948	Camphene	0.23	0.17	0.20	0.46	0.33	0.34
4	973	β-Phellandrene	2.51	1.46	1.56	1.97	1.30	1.06
5	975	Sabinene	0.79	0.58	0.56	1.34	1.08	0.87
6	994	β-Pinene	0.55	0.42	0.48	1.33	1.39	1.21
7	1020	a-Terpinene	0.08	0.08	0.02	0.05	0.07	ND
8	1034	D-Limonene	12.68	7.04	4.77	25.18	21.38	13.11
9	1047	Cyclofenchene	ND	0.01	ND	ND	0.01	ND
10	1058	b-Ocimene	ND	ND	ND	ND	0.01	ND
11	1064	γ-Terpinene	0.16	0.14	0.04	0.08	0.10	0.02
12	1090	Terpinolene	0.08	0.06	0.03	0.13	0.12	0.07
		Oxygenated monoterpenes						
13	1035	1,8-Cineole	ND	3.42	6.19	ND	ND	3.11
14	1071	cis-4-Thujanol	0.09	0.07	0.19	0.17	0.10	0.17
15	1097	trans-4-Thujanol	0.04	0.03	0.03	0.04	0.02	0.02
16	1103	Linalool	1.12	1.48	1.23	1.01	1.29	1.26
17	1122	4-Thujanol	ND	0.04	ND	ND	ND	ND
18	1123	γ-Terpineol	0.04	ND	ND	ND	ND	ND
19	1136	cis-Chrysanthenol	ND	ND	ND	0.03	ND	0.03
20	1142	(+)-2-Bornanone	ND	ND	0.03	ND	ND	0.02
21	1151	trans-Verbenol	0.19	0.21	0.16	0.25	0.32	0.31
22	1159	Citronellal	0.36	0.24	0.35	0.43	0.32	0.50
23	1166	Borneol	0.21	0.07	0.04	0.15	0.05	0.05
24	1170	Iso-neral	1.12	1.12	1.04	1.55	1.81	1.79
25	1177	4-Terpineol	0.49	0.47	0.13	0.16	0.14	0.04
26	1187	Iso-geranial	1.59	1.63	1.46	2.09	2.50	2.29
27	1190	α-Terpineol	0.60	0.68	1.32	0.44	0.51	1.00
28	1204	Carveol	0.06	0.04	ND	ND	0.02	0.07
29	1236	Nerol	0.46	0.06	ND	0.41	0.72	0.28
30	1241	Isopulegol	ND	ND	ND	0.06	0.03	ND
31	1251	β-Citral (Neral)	32.13	33.25	34.47	25.10	25.96	30.58
32	1268	Geraniol	1.40	3.07	0.25	1.30	1.75	0.35
33	1282	α-Citral (Geranial)	40.30	40.60	43.51	32.18	34.43	38.34
34	1420	Berbenone	ND	0.06	ND	ND	ND	ND
35	1454	Eugenol	ND	0.01	ND	ND	ND	ND
		Sesquiterpenes						
36	1336	Elixene	ND	ND	ND	ND	0.04	0.03
37	1373	α-Copaene	0.02	ND	ND	0.17	0.11	0.08
38	1413	β-Caryophyllene	0.06	0.04	0.05	0.52	0.60	0.52
39	1448	α-Caryophyllene	ND	ND	ND	ND	0.05	0.04
40	1448	Bicyclogermacrene	ND	ND	ND	0.04	ND	ND
41	1462	Isocaryophyllene	ND	ND	ND	ND	0.03	ND
42	1482	Aromandendrene	ND	0.01	ND	0.07	0.10	0.01
43	1522	d-Cadinene	ND	ND	ND	ND	0.02	0.03
		Oxygenated sesquiterpenes						
44	1575	Caryophyllene oxide	ND	0.02	0.04	ND	ND	0.06
		Others						
45	989	Sulcatone	0.47	0.36	0.45	0.32	0.29	0.33
46	1194	Methyl salicylate	0.08	0.06	ND	0.04	0.01	ND
47	1287	Bornyl acetate	0.20	0.26	0.16	0.17	0.27	0.11
48	1338	Nerol acetate	0.08	ND	0.08	0.13	ND	ND
49	1352	α-Terpinyl acetate	0.59	1.16	0.28	0.57	0.85	0.25
		Total oxygenated compounds	80.18	86.56	90.44	65.37	69.98	80.28
		Total non-oxygenated compounds	17.99	10.63	8.30	33.06	28.22	18.52
		Total	99.59	99.03	99.71	99.67	99.62	99.50
		Yield (%) *	1.35 ± 0.02 ^c,d^	1.44 ± 0.06 ^c^	1.25 ± 0.08 ^d^	1.86 ± 0.04 ^b^	2.27 ± 0.11 ^a^	1.80 ± 0.06 ^b^

HD: hydrodistillation, SFME: solvent-free microwave extraction, ND: not detected. ^#^ Calculated retention index (RI) relative to standard mixture of n-alkanes (C7–C40) on an HP-5 MS capillary column. * Yield with the same letter in the same row are not significant at *p* < 0.05, which are presented as the means ± standard deviation of the triplicate.

## Data Availability

Data are contained within the article.
